# Molecular Evolution and Epidemiological Characteristics of SARS COV-2 in (Northwestern) Poland

**DOI:** 10.3390/v13071295

**Published:** 2021-07-02

**Authors:** Karol Serwin, Andrzej Ossowski, Maria Szargut, Sandra Cytacka, Anna Urbańska, Adam Majchrzak, Anna Niedźwiedź, Ewa Czerska, Anna Pawińska-Matecka, Joanna Gołąb, Miłosz Parczewski

**Affiliations:** 1 Department of Infectious, Tropical Diseases and Immune Deficiency, Pomeranian Medical University in Szczecin, 71-455 Szczecin, Poland; karol.serwin@pum.edu.pl (K.S.); urbanska@pum.edu.pl (A.U.); 2 Department of Forensic Medicine, Pomeranian Medical University in Szczecin, 70-111 Szczecin, Poland; forensicmedicine1978@gmail.com (A.O.); maria.szargut@gmail.com (M.S.); cytackasandra@gmail.com (S.C.); 3 Independent Public Regional Hospital in Szczecin, 71-455 Szczecin, Poland; amajchrzak@spwsz.szczecin.pl (A.M.); ania.niedzwiedz@gmail.com (A.N.); czerska@spwsz.szczecin.pl (E.C.); pawinska@spwsz.szczecin.pl (A.P.-M.); asiak119@interia.pl (J.G.)

**Keywords:** phylogeny, clade, lineage, virus circulation, molecular tracing, SARS-Cov-2 outbreak in Poland

## Abstract

The emergence of severe acute respiratory syndrome coronavirus-2 (SARS-CoV-2) evolved into a worldwide outbreak, with the first Polish cases in February/March 2020. This study aimed to investigate the molecular epidemiology of the circulating virus lineages between March 2020 and February 2021. We performed variant identification, spike mutation pattern analysis, and phylogenetic and evolutionary analyses for 1106 high-coverage whole-genome sequences, implementing maximum likelihood, multiple continuous-time Markov chain, and Bayesian birth–death skyline models. For time trends, logistic regression was used. In the dataset, virus B.1.221 lineage was predominant (15.37%), followed by B.1.258 (15.01%) and B.1.1.29 (11.48%) strains. Three clades were identified, being responsible for 74.41% of infections over the analyzed period. Expansion in variant diversity was observed since September 2020 with increasing frequency of the number in spike substitutions, mainly H69V70 deletion, P681H, N439K, and S98F. In population dynamics inferences, three periods with exponential increase in infection were observed, beginning in March, July, and September 2020, respectively, and were driven by different virus clades. Additionally, a notable increase in infections caused by the B.1.1.7 lineage since February 2021 was noted. Over time, the virus accumulated mutations related to optimized transmissibility; therefore, faster dissemination is reflected by the second wave of epidemics in Poland.

## 1. Introduction

Coronaviruses (CoVs) are a family of large, up to ~30kb in length single-stranded RNA viruses. Four phylogenetically distinct groups (alpha, beta, gamma, delta) have been identified, including seven human coronavirus variants (hCoVs): the alpha-CoVs HCoVs-NL63 and HCoVs-229E, beta-CoVs HCoVs-OC43 and HCoVs-HKU1, severe acute respiratory syndrome-CoV (SARS-CoV), the Middle East respiratory syndrome-CoV (MERS-CoV), and lastly SARS-CoV-2 [1]. The emergence of the last one, identified in Wuhan, China, is responsible for the ongoing pandemic of coronavirus disease 2019 (COVID-19) and has initiated unprecedented efforts in studies on its molecular evolution, including whole-genome sequencing [2]. Coronaviruses possess lower mutation rates than other respiratory RNA viruses (a rate of ∼1.1 × 10^−3^ substitutions per site per year, corresponding to one substitution every ∼11 days); however, a high number of infections in the expanding epidemics translates into increasing interhost genomic diversity [3]. 

As of 22 June 2021, more than 2,041,359 complete genomes of SARS-CoV-2 were deposited in the Global Initiative on Sharing All Influenza Data (GISAID) database [4]. This extensive molecular surveillance of virus requires a straightforward approach to the classification of variant genetic diversity. Three main nomenclatures have been introduced for SARS-CoV-2, including Nextstrain clades [5], PANGO lineages (PANGO, Phylogenetic Assignment of Named Outbreak LINeages) [6], and GISAID groups [7]. While PANGO provides more detailed outbreak cluster information, the other two classifications offer broad geographical and temporal clade trends. A vast amount of molecular data enables real-time tracing of the pandemic evolution by investigation of the outbreaks and surveillance of the emergence of novel circulating strains. 

The emergence of the novel variants requires constant molecular surveillance, as molecular diversity results in evolution into variants of concern (VOC), with clear scientific evidence on the improved transmissibility, immune escape, or severity. This evolution may notably hinder efforts in the combat of SARS-CoV-2 pandemics [8]. Furthermore, expanding population immunity exerts novel selection pressures on the virus, further underscoring the importance of monitoring the vaccine, convalescent plasma, and immunoglobulin escape variants [9]. Novel virus strains are also classified as variants of interest and variants under monitoring, with preliminary data suggesting an increase in transmission risk or disease severity. Key VOCs currently circulating in Europe and worldwide are variants B.1.1.7 originating in the UK, which has dominated the EU epidemics in recent months, B.1.351 from South Africa, and P.1 first identified in Brazil, all with clear evidence on their increased transmissibility and severity [10,11]. 

All VOCs and most variants under surveillance harbor mutations within the spike protein-coding regions, allowing for optimized binding to the human angiotensin-converting enzyme receptor (ACE-2). Specific mutations, such as D614G, have been fixed in the circulating viral strains since the initial months of the pandemic, while others are common among the variants of increased virulence (e.g., N501Y, E484K) [12,13]. Additionally, deletions in the spike-coding regions, such as ΔH69/V70, were associated with the increased incorporation of spike into virions, which may act as a permissive factor allowing for the emergence of other deleterious mutations [14]. Studies on the impact of the mutations on virus evolution are ongoing, and continuously identify novel variants and mutations, with the key recent ones being L452R, E484Q, and T478K from Indian isolate B.1.617–VOC *Delta* and *Kappa* [15]. 

In Poland, the first confirmed case of the COVID-19 disease was registered on 3 March 2020 (https://www.gov.pl/web/coronavirus accessed on 22 June 2021). The epidemic has evolved in three waves so far. The first was a mild one observed in the spring of 2020, while the second and third waves have been associated with the high COVID-19 rates/100,000, significant mortality, and limited access to medical services. Recent increases in the number of new cases have been associated with the introduction of the B.1.1.7 variant into the population. So far, national totals as of 21 June 2021 were 2,878,840 cases of infections and 74,829 deaths which translate into <3% official COVID-19 mortality [16]. The country is scaling up the molecular detection and sequence-based identification efforts to provide up-to-date information on the population evolution of the virus; however, so far, no detailed phylogenetic study on the variant evolution and mutation emergence has been published. 

In the present study, we aimed to present molecular surveillance data on SARS-Cov-2 variant evolution in the first year of the pandemic in Poland, with the characteristics of spike protein mutations, based on sequences from Northwestern Poland supplemented with the GISAID data. Moreover, we performed evolutionary and epidemiological analyses to reflect the characteristics of the virus variants circulating at the country level, tracing the origin and the temporal population dynamics of SARS-CoV-2 in Poland.

## 2. Materials and Methods

### 2.1. Study Group

In this study, a dataset of locally obtained samples (159 cases) with nucleic acid amplification testing (NAAT)-confirmed SARS-CoV-2 infection was supplemented with a set of 1005 sequences (a total of 1164 sequences) from Poland available in the public GISAID database as of 1 March 2021. Clinical data were obtained from medical record reviews or collected by the sequencing laboratory in the process of clinical testing. 

### 2.2. SARS-CoV-2 Whole Genome Sequencing (WGS)

RNA was extracted using the MagMAX Viral/Pathogen Nucleic Acid Isolation Kit (Thermo Fisher Scientific (TFS), Vantaa, Finland), and the automated KingFisher Flex (TFS, Singapore) instrument for automated sample purification according to manufacturer instructions. Subsequently, the extracted RNA was quantified with TaqMan 2019-nCoV Assay Kit v1 and TaqMan 2019-nCoV Control Kit (TSF, Vilnius, Lithuania) on the Applied Biosystems QuantStudio 5 (TFS, Singapore) Real-Time PCR instrument. Next, the RNA was reverse transcribed to cDNA with the SuperScript VILO cDNA Synthesis Kit (TFS, Carlsbad, CA, USA) in an Applied Biosystems Veriti 96-Well Thermal Cycler (TSF, Singapore). Libraries were prepared manually as detailed in the Ion AmpliSeq Library Kit Plus (TSF, Carlsbad, CA, USA) user guide (MAN0017003) and Ion AmpliSeq SARS-CoV-2 Research Panel (MAN0019277). Finally, sequencing of the SARS-CoV-2 genomes was carried out on the Ion GeneStudio S5 System (TFS, Waltham, MA, USA). The reads were aligned with the reference genome (accession number: MN908947.3) in the Torrent Suite v.5.12.1 software (Euformatics Oy, Espoo, Finland). For the sequences mapping, several plugins were used: Coverage Analysis, Variant Caller, IRMA, and COVID19AnnotateSnpEff.

### 2.3. Sequence Data Sets

The MAFFT v.7.471 program [17] was used to align and remove sequences with more than 5% ambiguous letters. Following quality control, from the initial dataset of 1164 Polish SARS-CoV-2 sequences we selected 1106 high-coverage full-genome sequences including 122 local Northwestern Poland samples and 984 genomes obtained from GISAID. In the next step, the SARS-CoV-2 alignments were filtered, and sequences were masked following the script published by Nicola del Mario et al. [18]. For SARS-CoV-2 variant identification and mutation calling, we employed two lineage assignment tools: PANGOLIN v2.3.2 (https://github.com/cov-lineages/pangolin (accessed on 15 March 2021); referred to as PANGO) and NEXTCLADE v0.14.2 (https://github.com/nextstrain/nextclade (accessed on 15 March 2021); referred to as Nextstrain). Finally, for phylogeographic analyses, each West Pomeranian sequence was used as a query in a BLAST search (Basic Local Alignment Search Tool) against all the GISAD SARS-CoV-2 sequences. For every West Pomeranian isolate, ten of the most similar sequences were downloaded, and duplicates removed. As a result, a set of 376 sequences related to Polish Northwestern SARS-CoV-2 genomes was obtained. Quality checks of the final sequences and evaluation of genetic distance were performed in MEGAX software [19]. 

### 2.4. Phylogenetic and Phylodynamic Analyses

All phylogenetic trees were generated with IQ-Tree v2.0.5 [20] using the maximum likelihood (ML) method with approximate likelihood ratio test (aLRT) and ultrafast bootstrap with 1000 replicates. The GTR+F+G model with four gamma categories was selected as optimal for the analyzed dataset using ModelFinder accuracy estimates [21]. All trees were visualized in the Interactive Tree of Life (iTOL) [22]. After TempEst v.1.5.3 [23] analysis, the SARS-CoV-2 phylogenies exhibited a moderate association between genetic distances and sampling dates and were suitable for phylogenetic molecular clock analysis in Bayesian Evolutionary Analysis by Sampling Trees (BEAST). A large residual point scatter from the regression line suggested that a relaxed molecular clock model should be most appropriate for subsequent analysis. Different coalescent tree priors for identified clade 1–3 sequences were separately implemented in the BEAST v.1.10.4 software package [24]. For the time-scaled analysis, the uncorrelated relaxed clock model with an underlying lognormal distribution (UCLN) and tree prior of coalescent Bayesian skyline growth population with five groups piecewise-constant model was used. As identified by ModelFinder, we used a multiple continuous-time Markov chain (MCTMC) GTR+F+G4 model. Five hundred million Markov chain Monte Carlo (MCMC) runs with sampling every 10,000 steps were computed and processed in two independent replicates of the same inference [25]. LogCombiner was used for combining the output from multiple runs, and results were visualized and checked in Tracer v1.8 [26]. The effective sampling size values (ESS) were 200 or more, indicating adequate convergence. 

The Bayesian birth–death skyline model (BDSKY) [27] was implemented in BEAST v2.62 [28] to estimate changes in the effective reproductive number (R_e_) for three clades separately. For heterochronous data, the Birth Death Skyline Serial prior with UCLN distribution was selected under a GTR+G4 substitution model based on ModelFinder selection. The evolutionary rates for each clade were based on the slope of root-to-tip plots assigned in TempEst. A lognormal distribution with a mean of 0 and standard deviation of 1.0 for *R_e_* was chosen, and the dimension of the parameter was selected to be five. The rate to become uninfectious (δ) had a normal distribution with a mean of 48.7 and a standard deviation of 15. These values reflect the inverse of the time of infectiousness and were estimated by Li Q. et al. [29]. The sampling proportion (ρ) prior was assessed with the alpha parameter set to 1 and beta to 9999. The origin of the epidemic was approximated with normal prior using a mean of 0.25 and standard deviation of 0.05 units per year, as described elsewhere [30]. MCMC ran for at least 200 million generations and was sampled every 50,000 steps. The ESS value reads were diagnosed using Tracer, and values above 200 indicated sufficient sampling.

### 2.5. Statistics and Visualization

Statistical comparisons were performed with Fisher’s exact and X^2^ tests for nominal variables, as needed. The confidence intervals (CI) were marked as appropriate. Statistical calculations were made with commercial software (Statistica v13. Statasoft, Warsaw, Poland). The R (4.0.2.) platform [31] was performed with packages including MASS [32] for time trends and logistic regression, CORRPLOT [33] for Spearman Rank test, and VCD [34] to visualize the two-way contingency plots.

## 3. Results

### 3.1. Prevalence of SARS-CoV-2 Variants

**Figure 1:**
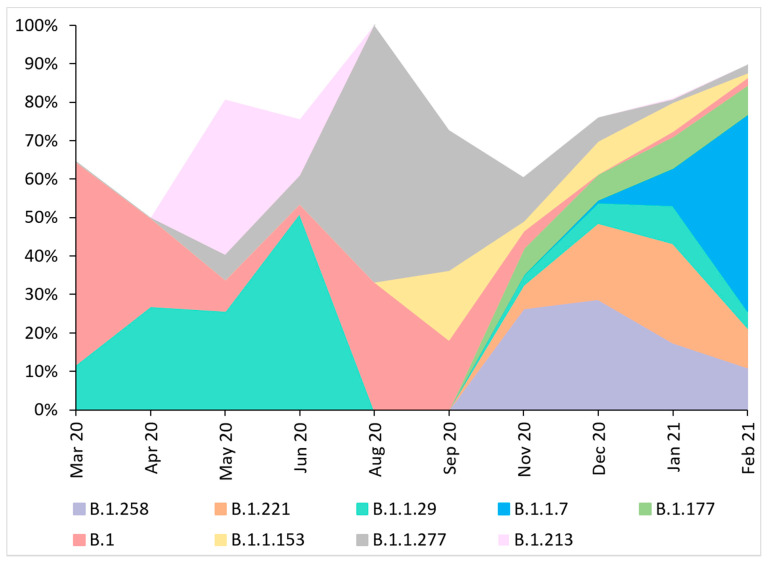
Lineage size breakdown of Poland genomes collected each month. Distribution of major nine SARS-Cov-2 lineages circulating in Poland between March 2020 and February 2021, estimated based on whole-genome sequencing. No data on the sequences collected in July and October 2020 were available. Displayed codes indicate virus variants based on PANGO classification. During the first month of analysis, the dominant virus variant was lineage B.1 (20A), with 27 cases observed in March 2020 (52.94%), and lasted during the entire period of sampling. In April and June 2020, variant B.1.1.29 (20A) dominated in 20 (27.03%) and 21 (51.22%) of sequenced isolates, respectively. In May, lineage B.1.213 (20A) appeared, which accounted for 40.32% (n = 25) of cases. In August and September, the most common virus type was B.1.1.227 (20B), at 66.67 (n = 3) and 36.36% (n = 4), respectively. In the next two months, the B.1.258 (20A) variant dominated (n = 30 (27.27%) for November and n = 47 (29.19%) in December). In January 2021, the main virus strain was B.1.221 (20A) (n = 115 (26.08%)). February showed the highest contribution of variant B.1.1.7 (20I/501Y.V1) with 79 cases (51.97%).

The predominant variant in the analyzed dataset was PANGO B.1.221/Nextstrain 20A (n = 170, 15.37%). In 166 sequences (15.01%), the B.1.258 (Nextrain 20A) variant was identified, followed by 127 cases (11.48%) of B.1.1.29 (Nextsrain 20B) lineage, and 123 (11.12%) sequences of B.1.1.7 (Nextrain 20I/501Y.V1) variant. Nineteen (1.72%) sequences were identified as Nextstrain 19A clade and a single isolate (0.09%) as 19B, reflecting the events of virus introduction to Poland directly from China. All the remaining sequences (n = 1086, 98.19%) were clade 20A, originating in Europe. Detailed characteristics and differences in the lineage distribution between Northwestern Poland and the rest of the country and dynamics over time are presented in the Supplementary Material. Analyzed molecular data indicate that from March 2020 to February 2021 in Poland, notable differences in the variant distribution over time may be observed, with increasing variant diversity since November 2020 (Figure 1).

### 3.2. Phylogenetic Analysis of SARS-CoV-2 Genomes

**Figure 2:**
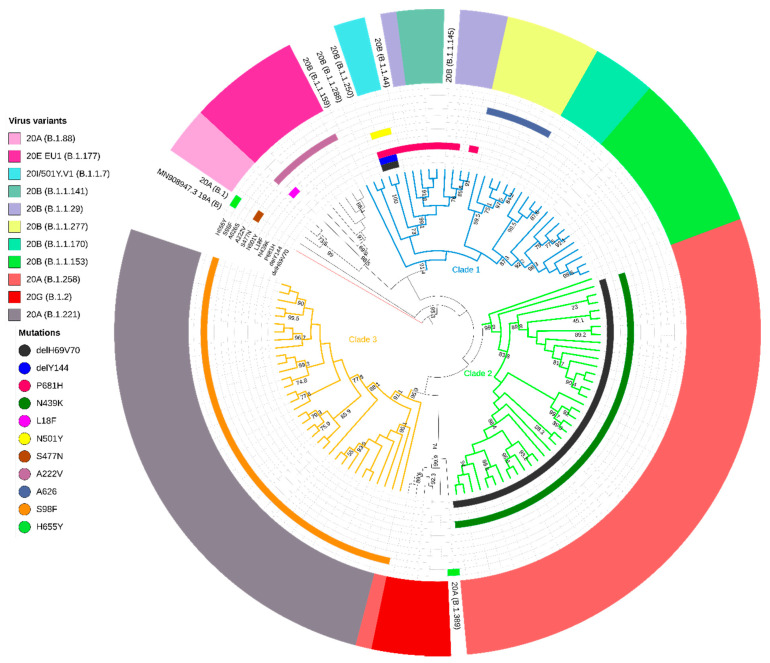
Maximum likelihood (ML) phylogeny of West Pomeranian SARS-CoV-2 whole-genome sequences (n = 122)**.** Branches of the three main clades are colored in blue, green, and orange. Specifically, the most common mutations in spike are highlighted: ΔH69/V70, ΔY144, P681H, N439K, L18F, N501Y, S477N, A222V, A626S, S98F, and H655Y. Inner rings indicate the presence or absence of the deletion and amino acid variants. The multicolored outer ring shows virus variants in the West Pomeranian region together with the Wuhan reference sequence (red branch, GenBank accession no. MN908947.3). Virus variants were assigned in Nextstrain (and PANGO lineage) classification.

Phylogenetic analysis revealed that 105 (86.07%) sequences from the West Pomerania region formed three main clades with aLRT support >90% (Figure 2). We performed phylogenetic reconstruction of the sequences and the ancestry to identify clade-defining mutations (Table 1). For the purpose of this study we named these monophyletic groups as Clade 1 (n = 34 genomes, 27.87%), Clade 2 (n = 37, 30.33%), and Clade 3 (n = 34, 27.87%). Nextstrain and PANGO lineage identification methods were used to assign the sequence groups. Genomes in Clade 1 were genetically diverse and classified as lineages 20B and 20I/501Y.V1 by Nextstrain and 11 PANGO variants (B.1.1.*), with B.1.1.153 (n = 10, 8.20%) being the most common. All of the isolates in Clade 2 were Nextstrain 20A clade and PANGO B.1.1.258 lineage, while sequences from Clade 3 belonged to the 20A Nextstrain group and PANGO B.1.1.221 variant. For each clade, we identified a set of ORF1a, ORF1b, ORF3a, ORF7b, S, and N mutations (Table 1). The mean number of base substitutions per site within the clade (6.58 × 10^−4^, SEM = 4.23 × 10^−5^) versus mean inter-clade variability (1.32 × 10^−3^, SEM = 1.04 × 10^−4^) showed the higher intra-group sequence similarity, as shown in the inferred phylogenetic tree (estimates of clade average evolutionary divergence presented in Supplementary Table S1).

**Figure 3:**
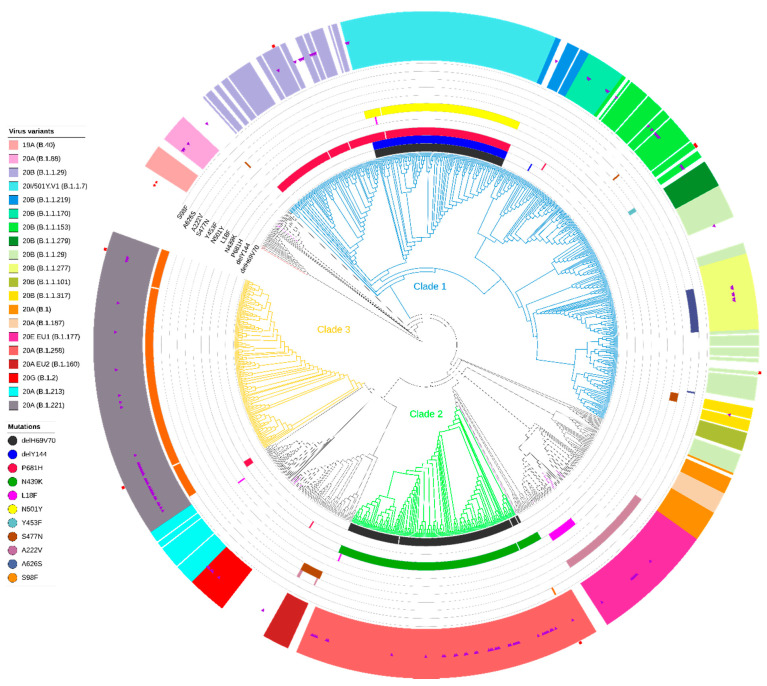
Maximum likelihood (ML) phylogeny of polish SARS-CoV-2 whole-genome sequences (n = 1106). Branches of the three main clades are colored in blue, green, and orange. Specific mutations in spike are highlighted: ΔH69/V70, ΔY144, P681H, N439K, L18F, N501Y, Y453F, S477N, A222V, A626S, and S98F). Inner rings indicate the presence or absence of the deletion and amino acid variants. The multicolored outer ring shows the presence of the most common virus variants in Poland. The purple triangles mark the 122 sequences from the West Pomeranian region. The red stars indicate Wuhan reference sequences (GenBank accession no. MN908947.3 and GISAID accession no. EPI ISL406801). Polish sequences (n = 984) were obtained from the GISAID database (accessed 1 March 2021). Red squares indicate seven sequences that did not follow the clade-defining mutations pattern. Virus variants were assigned in Nextstrain (and PANGO lineage) classification.

When analyzed in a broader national context with the remaining 984 Polish sequences included into the dataset, the three clades identified above contained 74.41% (n = 823) of sequences, with clade 1 (B.1.1*) consisting of 500 (45.21%) sequences belonging to the three Nextstrain clades (20B (n = 364, 32.91%), 20I/501Y.V1 (123, 11.12%), 20D (13, 1.18%)) (for details see Supplementary Material). Clade 2 contained 160 (14.47%) sequences (only Nextstrain 20A, PANGO B.1.1.258 variant), while for Clade 3 we reported 163 (14.74%) sequences which corresponded to Nextstrain clade 20A and the PANGO B.1.1.221 variant (Figure 3). 

**Figure 4:**
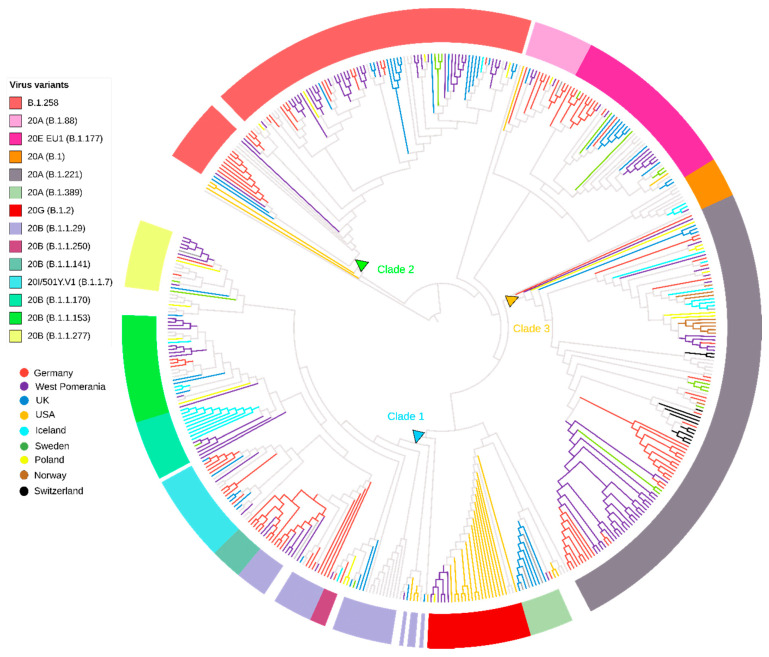
Phylogenetic tree of West Pomeranian SARS-CoV-2 sequences (n = 122) with an international context. Maximum likelihood tree of West Pomeranian SARS-CoV-2 sequences and a subset of BLAST sequences (n = 376) from GISAID database. Thirty countries of isolates’ origin have been identified. For eight countries that had a minimum of four (>1%) distinguished sequences, branches are colored. Multicolor rings correspond to the PANGO and Nextstrain virus classification. The blue, green, and orange triangles mark the node origins of the Clades 1–3.

Furthermore, we performed a phylogenic analysis of the genomes related to those observed in Northwestern Poland using the GISAID database as a reference. A BLAST search indicated 376 sequences with high homology to our dataset, used for phylogenetic reconstruction (Figure 4). The SARS-CoV-2 strains from Clade 1 (lineages B.1.1.*) in Northwestern Poland were intermixed mainly with variants from Germany (n = 29, 7.71%) and, expectedly, the United Kingdom (n = 18, 4.79%). Clade 2 (lineage B.1.258) clustered dominantly with German (n = 23, 6.12%) and British sequences (n = 16, 4.26%), while Clade 3 (lineage B.1.221) was related mainly to German (n = 38, (10.11%) and Swiss (n = 11, 2.93%) SARS-CoV-2 genomes (details described in Supplementary Material). 

### 3.3. Genetic Variability of the SARS-CoV-2 Spike Protein

This section examines the variability of the SARS-CoV-2 spike protein-coding region in the analyzed dataset. In total, we identified 21 mutations, of which 11 were found in more than 5% of the sequences (Figure S4). D614G substitution was fixed (97.47%) in the circulating viruses in Poland. 

**Figure 5:**
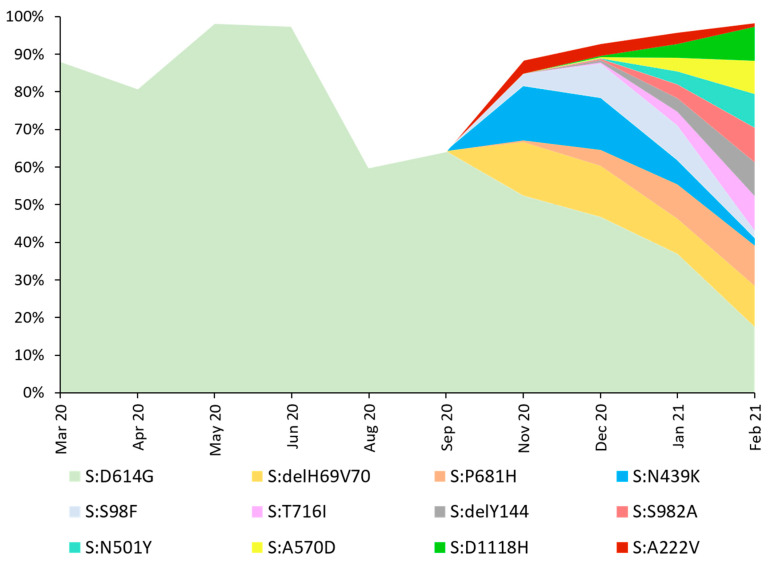
Distribution of SARS-CoV-2 spike mutation in Poland over time. Contribution of individual mutations of the spike protein genetic diversity between March 2020 and February 2021 identified in SARS-Cov-2 strains. No data on the sequences collected in July and October were available. Mutations found in more than 5% of the total analyzed isolates are presented.

Spike protein H69V70 deletion was the second most common mutation, observed in 279 (25.23%) sequences. Genetic variability of the SARS-CoV-2 and number of spike mutation containing variants increased rapidly since September 2020, with additional accumulation of P681H, N439K, S98F, during the second wave of SARS CoV-2 epidemics in Poland (Figure 5, Tables S1 and S2). 

**Figure 6:**
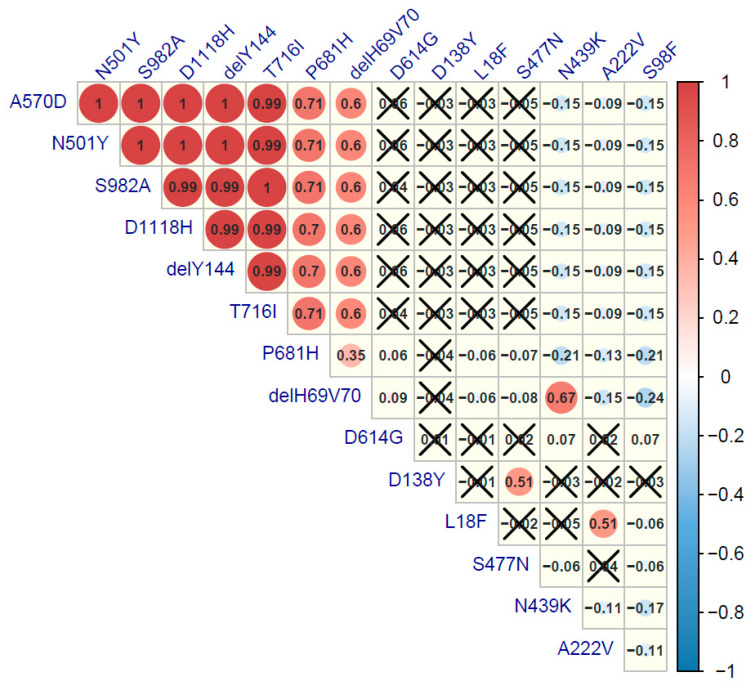
Correlation plot of major spike mutations in SARS-Cov-2 isolates from Poland. Positive correlations are displayed in red and negative correlations in blue. The color intensity and the circle size are proportional to the correlation coefficient values. Spearman’s rank test was performed, and a *p*-value ≤ 0.05 was considered as significant. Correlation coefficient values with greater *p*-value are cross-marked.

The frequencies of the 14 most frequent sequence changes were compared using the Spearman’s correlation rank test to reflect the co-existence of the mutations in the analyzed genomes. Six substitutions and two deletions were strongly correlated (Figure 6). Co-existence of ΔH69V70, ΔY144, P681H, T716I, S982A, A570D, N501Y, and D1118H was the signature for the B.1.1.7 (20H/501Y.V1) variant. Additionally, the other three patterns of mutation co-evolution were confirmed. The first one was ΔH69V70 and N439 in lineage B.1.258 (20A), then D138Y with S477N observed for B.1.1.317 (20B), and finally A222V and L18F in the B.1.177 (20E EU1) strains.

### 3.4. Temporal Trends for Spike Mutation Frequency

**Figure 7:**
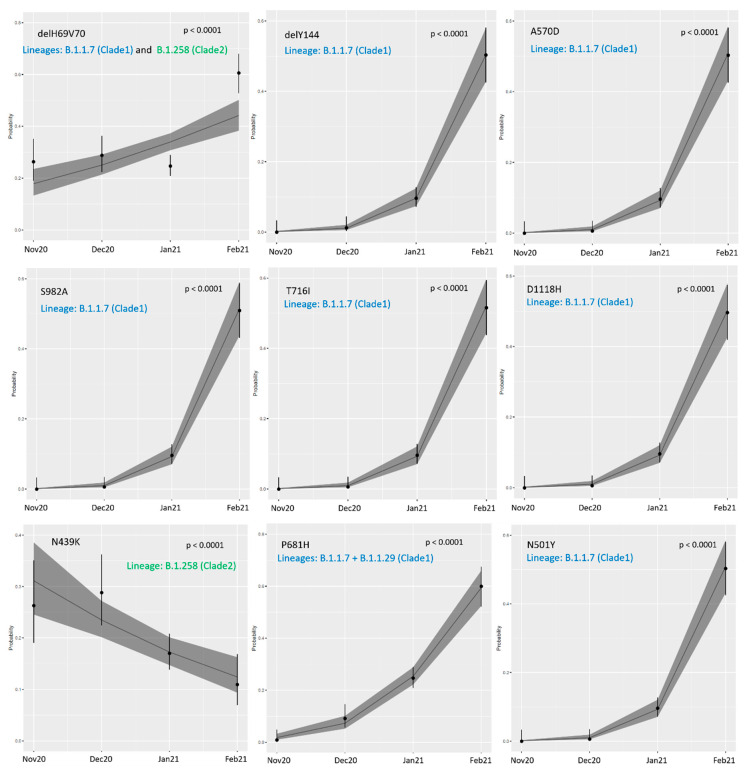
Logistic regression estimates for time trends between November 2020 and February 2021 for spike protein mutations identified in Polish isolates. Colored font describes lineages in which mutations was found.

For the analyses of time trends related to spike mutation frequency, samples obtained before November 2020 were excluded due to low genetic diversity. The proportion of eight analyzed most common missense mutations in the spike protein-coding region increased significantly over time (Figure 7 and Table 2). Notably, ΔH69V70 frequency rose from 26.31% in November to 60.65% in February (OR: 1.54, 95% CI: 1.30–1.83, *p* < 0.0001); for the ΔY144 mutation this increase was from 1.23% in December to 51.32% in February; and for the P681H mutation from 0.88% in November to 60% in February (OR: 9.52, 95% CI: 6.46–14.35, *p* < 0.0001 and OR: 4.30, 95% CI: 3.32–5.67, *p* < 0.0001, respectively). Additionally, for the T716I and S982A variants, substitution frequencies increased over time (respectively, from 0.88% in December to 51.61% in February (OR: 10.59, 95% CI: 7.13–16.11, *p* < 0.0001) and from 0.88% in December to 50.97% in February (OR: 10.53, 95% CI: 6.97–15.72, *p* < 0.0001). 

Two mutations, A570D and N501Y, were in perfect linkage; therefore, the increase in incidence from 0.88% in December to 51.32% in February (OR: 10.12, 95% CI 6.83–15.37, *p* < 0.0001) was the same. The last notable increasing trend was detected for D1118H, from 0.88% in December to 49.68% in February (OR: 9.90, 95% CI 6.83–15.01, *p* < 0.0001). Interestingly, only the frequency of N439K dropped, from 26.31 to 10.97% (OR: 0.68, 95% CI 0.57–0.82, *p* < 0.0001) in the analyzed months. Of note, when excluding the B.1.1.7 variant from temporal trends analysis, only four mutations (delH69V70, P681H, S98F, A222V) had a significantly different frequency from November 2020 until February 2021 (see Figure S5 and Table S4).

### 3.5. Phylodynamic Analysis of the Polish Dataset

The mean tMRCA (time to most recent common ancestor) was estimated for Clade 1 (lineages B.1.1.*) to 27 January 2020 (95% highest posterior density—HPD; from 12 December 2019 to 1 March 2020); for Clade 2 (lineage B.1.258) to 18 March 2020 (95% HPD between 24 September 2019 and 13 July 2020); and for Clade 3 (lineage B.1.221) to 16 June 2020 (95% HPD from 11 March 2020 to 3 September 2020).

**Figure 8:**
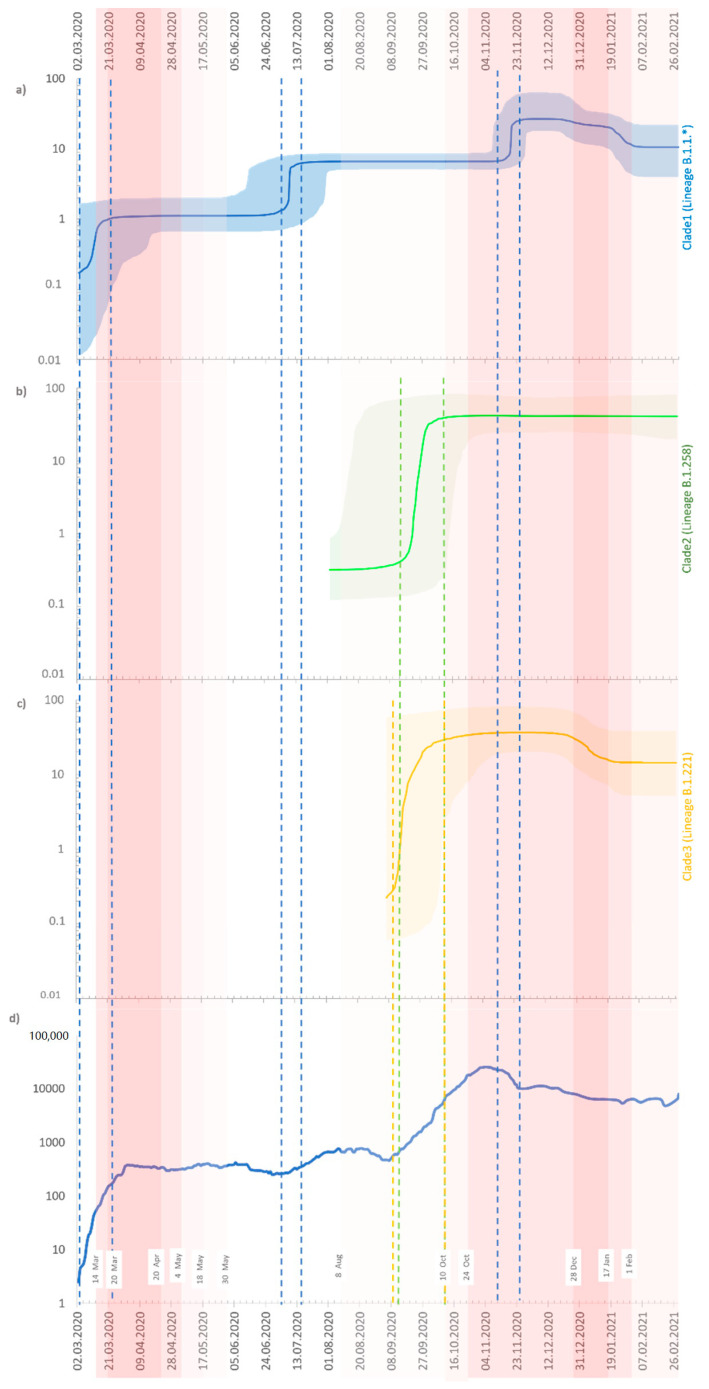
Bayesian skyline plot of the Polish SARS-CoV-2 outbreaks. (**a**) Clade 1 (lineages: B.1.1.*—for details see Supplementary Materials); (**b**) Clade 2 (lineage: B.1.258); (**c**) Clade 3 (lineage: B.1.221); and (**d**) registered cases of COVID-19 in Poland (https://www.gov.pl/web/coronavirus, accessed on 22 June 2021). For (**a**–**c**), the Y-axis indicates effective population size (N_e_), and the X-axis shows the time. The thick solid line represents the median value of the estimates, and the shaded area the 95% HPD (highest posterior density). For (**d**), four different red color intensities exhibit the four-stage restrictive measures implemented by the government at the country level. White rectangles indicate dates of introduction/abolition of the restrictions.

The Bayesian skyline plot (BSP) of the Clade 1 sequences shows three intervals with an increase in the number of infections (Figure 8a). First, at the beginning of the pandemic between 2 March 2020 and mid-March 2020. In this period, infections caused by variants B.1 and B.1.1.29 were dominant. Second, with exponential growth in the first half of July 2020 with an increasing prevalence of B.1.1.227. Finally, between 6 and 24 November 2020 was noted the high incidence of the B.1.1.29 lineage. For Clade 2 (lineage B.1.258), the BSP analysis revealed one timeframe with a rapid extension between 12 September 2020 and 1 October 2020 when it reached a plateau (Figure 8b). The last group of isolates—Clade 3 (lineage B.1.221)—exhibited exponential growth from 5 September 2020 to 1 October 2020, continuing at a high average until the end of the sampling time (Figure 8c).

**Figure 9:**
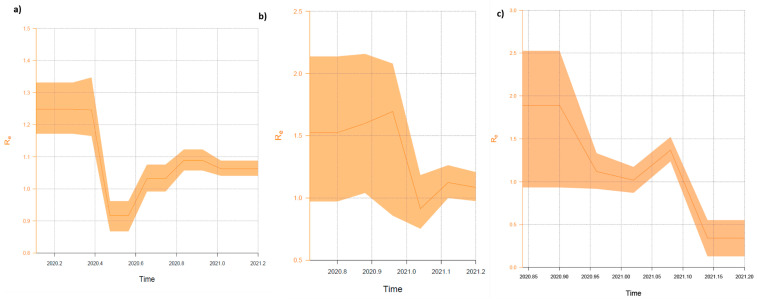
Birth–death skyline plot (BDSKY) of the Polish SARS-CoV-2 outbreaks. (**a**) Clade 1 (lineages: B.1.1.*—for details see Supplementary Materials); (**b**) Clade 2 (lineage: B.1.258); and (**c**) Clade 3 (lineage: B.1.221). R_e_ estimates obtained using the BDSKY model over five equidistant intervals. The curves and the orange shaded areas show the mean R_e_ values and the 95% confidence intervals. The Y axes indicate R values, and X axes time in years.

The effective reproduction number (R_e_) estimates of Clade 1 showed complex phylodynamics, indicated by two declining phases and two growing frames (Figure 9a). The mean value ranged from 1.25 (95% HPD 1.17–1.33) since the origin of the epidemic to 0.92 (95% HPD 0.87–0.96) in June–August 2020. In mid-August 2020, the curve started to grow until 1.089 (95% HPD 1.06–1.12) in November–December 2020 and finally reclined to 1.06 (95% HPD 1.04–1.09) at the end of sampling. For Clade 2, the curve started to grow in the second half of October 2020 and reached the value of 1.52 (95% HPD 0.97–2.14) in December 2020 (Figure 9b). After the new year, the value of R_e_ fell to its lowest level of 0.91 (95% HPD 0.76–1.04) and rose again to 1.08 (95% HPD 0.98–1.21) in February 2021. During the sampling period, R_e_ estimates for Clade 3 had the highest values. At the beginning of November 2020, R_e_ was 2.16 (95% HPD 1.74–2.64), then showed a fast decrease to 1.02 (95% HPD 0.87–1.17) at the end of January 2021. In February 2021, the R_e_ value peaked at 1.37 (95% HDP 1.24–1.52) and then dropped to 0.34 (95% HDP 0.13–0.55) in the first week of March 2021 (Figure 9c).

## 4. Discussion

Following the introduction of the novel virus into the population, surveillance studies assessing molecular evaluation and diversity remain of primary importance. Since the emergence of the SARS-CoV-2 pandemic, the expanding use of genomic technologies has unprecedently allowed the rapid and continuous update of the phylodynamic evolution of this virus [35]. The emergence of the novel variants and mutations affecting viral transmissibility and pathogenicity require constant phylogenetic updates to inform public responses and vaccine studies [36]. Novel variants may increase the R_0_ due to spike protein mutations with the modeling data suggesting 56–75% higher transmissibility compared to the previously circulating strains (https://www.ecdc.europa.eu/en/publications-data/covid-19-risk-assessment-spread-new-variants-concern-eueea-first-update accessed on 22 June 2021). In this study, we present novel data on the sequence evolution, mutation patterns and phylodynamics of SARS-CoV-2 from both the regional (Northwestern Poland) and national perspective, including temporal reconstructions.

We report a significant change in the virus characteristics over time, with the dynamic expansion of genetic variability, observed both as the increase in strain diversity and number of spike mutations, coinciding with the beginning of the second wave of epidemics in Poland observed from September 2020. In this period, the seven-day average in the country exceeded 20,000 cases with >800 cases per 100,000 during the peak of the wave; however, molecular detection of active infection was focused on symptomatic cases, which resulted in underreporting of the morbidity (https://covid19-surveillance-report.ecdc.europa.eu/ accessed on 22 June 2021), underscoring the importance of the presented research from the phylodynamic perspective with the added value of the presented coalescent and birth–death models [37].

Interestingly, the trees’ root includes local and other Polish sequences closely related to the 19A and 19B strains and the Wuhan-obtained reference. This may reflect the early introduction of the virus to Poland within the short time of virus entry to Europe. Similar phenomena were observed in Italy and Germany [38]. Our analysis shows that subsequently, three genetic lines of the SARS-CoV-2 molecular evolution have emerged, with the largest clade (Clade 1, lineages B.1.1.*) being the most diverse genetically, with a significant proportion (24.6% for this clade) of the VOC B.1.1.7 variant characterized by a signature combination of the ΔY144, N501Y, A570D, S982A, D1118H, and T716I spike mutations. This variant, however, became dominant in early 2021, which is in line with observations observed in other countries—Germany, the United Kingdom, and Denmark [10]. Interestingly, two genetically convergent strains (Clade 2 and 3) classified as B.1.258 and B.1.221 were responsible for almost 1/3 (29.2%) of the remaining infections in the analyzed period, with Clade 2 characterized by the conjunction of two spike mutations, ΔH69_V70 and N439K. It was previously suggested that ΔH69_V70 is associated with the increased transmissibility via spike incorporation into virions, and may be regarded as a “permissive mutation”, enhancing infection and allowing tolerance of the immune escape mutations related to the loss of replicative capacity. In comparison, N439K increases the binding affinity to ACE2 and may be responsible for the immune escape from the convalescent sera and monoclonal antibodies [39]. It has been indicated before that N439K co-occurs with ΔH69_V70 in the PANGO B.1.258 clade, exactly as confirmed in the presented phylogenetic analysis. In August 2020, this variant was present mainly in Ireland. It began to spread to Central Europe, with a high number of infections in the Czech Republic (November 2020) and Slovenia (December 2020/January 2021). In Poland, the peak of infection caused by the B.1.258 lineage was recorded in January 2021 (see Supplementary Figure S5) [40,41]. This lineage was probably introduced to Western Pomerania not from Southern Europe but from Germany or Nordic countries (Figure 4), where B.1.258 lineage was also noted at a moderate frequency (http://covarants.org/variants/S.N439K accessed on 22 June 2021). Clade 3 was the PANGO B.1.221 (Nextstrain 20A) strain with S98F mutation. This variant was found across Europe but mostly in Belgium, Netherlands, and United Kingdom (https://covariants.org/variants/S.S98F accessed on 22 June 2021). In the Benelux countries, this variant was reported frequently since the beginning of August 2020 (see Supplementary Figure S5) and is still circulating. The Netherlands and UK have the third- and first-largest representation of Polish emigrants in Europe (https://stat.gov.pl/en/ accessed on 22 June 2021) and likely have links with migration- and travel-related introduction of infections.

As presented, during the first wave of the SARS-CoV-2 epidemic in Poland the virus was less diverse genetically, with practically only a D614G spike mutation observed in the analyzed genomes. On the other hand, in the second wave of pandemics observed in autumn 2020, the molecular diversity of the virus has increased in line with an explosive number of cases and significant mortality. In this period, we report an expansion of more virulent variants such as B.1.1.7 VOC and strains not associated with the increased transmissibility per se but containing the described above ΔH69_V70, N439K, or P681H mutations. From November to the end of the analysis, the frequencies of the spike mutations increased by several folds, most likely reflecting the increasing dynamic of the infection in the population and a high number of circulating viral strains in the susceptible population. As shown, this increment was associated with the increasing prevalence of B.1.1.7, which naturally contains some of the spike mutations, the accumulation of deletions, and other substitutions in non-VOC strains. A decrease in the prevalence of the N439K variant was the exception. This was most likely due to the expansion of the B.1.1.7 variant, which does not contain N439K substitution, and a smaller proportion of the B.1.258 infections in early 2021. This was also reflected by the decrease in ΔH69_V70 frequency in non-B.1.1.7 and confirmed by the phylodynamic analyses birth–death skyline plot of Cluster 2 and 3, indicating drop in the R_e_ in the last months of the analysis.

As expected and suggested by the previous studies, D614G spike mutation has become fixed in the observed sequences as this variant was associated with in early sequences with transmission advantage and higher SARS-CoV-2 viral loads [42]. The second most common mutation is the ΔH69_V70 deletion. Further studies are required to indicate if this mutation will also predominate in the circulating strains, but it is also present in VOC B.1.1.7, it can be assumed that its frequency will continue to increase.

When analyzing the clustered Northwestern sequences from Poland, the highest homology with German sequences was observed for all identified clades, which is understandable due to the geographic location of the region but may importantly confirm the international cross-border spread of the SARS-CoV-2 between adjacent territories. Of note, in East Germany, the three main lineages, B.1.177, B.1.258, and B. 1.221, from the second wave (between August to October 2020) had been circulating, representing a very similar pattern to West Pomeranian isolates [43].

In the phylodynamic analyses, tMRCAs were in line with the epidemic course in Poland and support the observation of the increasing genetic diversity of the circulating virus. Moreover, the effective reproductive number estimated for March 2020 was greater than 1, which suggests the spread of the virus before the first confirmed COVID-19 isolate was collected. Skyline plots for all clades closely reflect the observed epidemiology, with peaks of cases seen from October/November 2020, explaining an increase in the number of infections in the same period. The values of R_e_ confirmed that B.1.1.* (Clade 1) was responsible for the spread of the first wave of infections in Poland. Furthermore, during the second wave of epidemics, all three clades expanded, with a high increment in variants B.1.221 (Clade 3) and B.1.258 (Clade 2). Restrictive measures introduced in the period from mid-October 2020 to mid-January 2021 reduced and stabilized the infections observed during the second wave of the epidemic.

The principal limitation of the study was the number and time frame of collected isolates from the West Pomerania region and analyzed GISAID sequences. They represent less than 2‰ of recorded SARS-CoV-2 infections in Poland. Nevertheless, the number of samples was substantial, allowed reliable phylogenetic analysis, and inferred population genetics. The scale of the epidemic excludes molecular surveillance of each isolate.

To conclude, continuous tracing of emerging virus lineages should be focused on variants of interests and variants of concern and the evolution of spike mutations. Phylodynamic studies identify the introductory events with subsequent spread of the virus and its divergence into clades. Increasing molecular variability during the second wave of pandemics in Poland might have resulted in the number of cases not only related to expanding infections with VOC B.1.1.7. Additionally, expansion of the variants bearing mutations related to optimized transmissibility and potentially higher virulence might have contributed to the epidemic waves. Continuous surveillance allows follow-up of virus evolutionary variability and the risks associated with the emergence of new variants.

## Figures and Tables

**Table 1:** Clade-defining mutations in the analyzed dataset. Positions are listed according to the reference genome (MN908947.3). Syn—synonymous mutation.

Gene	ORF1a:		ORF1b:					S:			ORF3a:		ORF7b:	N:
Position	7767	12988	15324	15598	17104	18028	20268	21855	22879	24910	25505	25996	27800	28883
Clade 1	T	G	C	G	C	G	A	C	C	T	A	G	C	C
Clade 2	C	T	C	A	T	T	G	C	A	C	A	G	A	G
Clade 3	T	G	T	G	C	G	A	T	C	T	G	T	C	G
Protein	I2501T	M4241I	Syn	V711I	H1213Y	A1521S	Syn	S98F	N439K	Syn	Q38R	V202L	Syn	G204R

**Table 2:** Time trends for missense mutations in spike protein.

Spike Mutation	Frequency in Analyzed Dataset n = 1106				OR	95% CI	*p*
	Nov 20	Dec 20	Jan 21	Feb 21			
delH69V70	30 (26.31%)	47 (29.19%)	110 (24.66%)	94 (60.65%)	1.54	1.30–1.83	<0.0001
delY144	0	2 (1.23%)	43 (9.75%)	78 (51.32%)	9.52	6.46–14.35	<0.0001
P681H	1 (0.88%)	15 (9.20%)	110 (24.66%)	93 (60.00%)	4.30	3.32–5.67	<0.0001
T716I	0	1 (0.88%)	43 (9.64%)	80 (51.61%)	10.59	7.13–16.11	<0.0001
S982A	0	1 (0.88%)	43 (9.64%)	79 (50.97%)	10.35	6.97–15.72	<0.0001
A570D	0	1 (0.88%)	43 (9.64%)	78 (51.32%)	10.12	6.83–15.37	<0.0001
N501Y	0	1 (0.88%)	43 (9.64%)	78 (51.32%)	10.12	6.83–15.37	<0.0001
D1118H	0	1 (0.88%)	43 (9.64%)	77 (49.68%)	9.90	6.68–15.01	<0.0001
N439K	30 (26.31%)	47 (29.19%)	76 (17.23%)	17 (10.97%)	0.68	0.57–0.82	<0.0001

ΔH69V70 was found in lineage B.1.1.7 (Clade 1), and in B.1.258 (Clade 2). ΔY144, T716I, S982A, A570D, N501Y, and D1118H was a signature mutation of B.1.1.7 (Clade 1) lineage. P681H was related to B.1.1.7 and B.1.1.29 (Clade 1) lineages. Finally, N439K persisted in the B.1.258 (Clade 2) lineage.

## Data Availability

The sequences used in this work have been deposited in the GISAID and can be found under the appropriate IDs: EPI_ISL_2631232–EPI_ISL_2631325, and EPI_ISL_2650471–EPI_ISL_2650498.

## Supplementary Materials

The following are available online at https://www.mdpi.com/article/10.3390/v13071295/s1, Figure S1: Prevalence of main SARS-CoV-2 lineages in Poland, Figure S2A and B: Two-way contingency plots of differences in Sars-CoV-2 variant prevalence by analyzed region, Figure S3: Frequency of major Spike mutation identified in Polish Sars-CoV-2 strains, Figure S4: Logistic regression estimates for time for Spike protein mutations identified in Polish isolates excluding sequences with B.1.1.7 variant, Figure S5: Local lineage dynamics for B.1.1.7 (Clade 1), B.1.221 (Clade 3) and B.1.258 (Clade 2) virus variants, Table S1: Estimates of average evolutionary divergence over West Pomeranian sequence pairs within and between Clades, Table S2: Differences in Sars-CoV-2 variants prevalence by analyzed region, Table S3: Differences in mutations of Sars-CoV-2 spike protein prevalence by analyzed region, Table S4: Time Trends for missense mutations in Spike Protein in non-B.1.1.7, and Supplementary data.

## References

[1] Payne S. (2017). Family Coronaviridae. Viruses.

[2] Wu D., Wu T., Liu Q., Yang Z. (2020). The SARS-CoV-2 outbreak: What we know. Int. J. Infect. Dis..

[3] Duchene S., Featherstone L., Haritopoulou-Sinanidou M., Rambaut A., Lemey P., Baele G. (2020). Temporal signal and the phylodynamic threshold of SARS-CoV-2. Virus Evol..

[4] Elbe S., Buckland-Merrett G. (2017). Data, disease and diplomacy: GISAID’s innovative contribution to global health. Glob. Chall..

[5] Hadfield J., Megill C., Bell S.M., Huddleston J., Potter B., Callender C., Sagulenko P., Bedford T., Neher R.A. (2018). Nextstrain: Real-time tracking of pathogen evolution. Bioinformatics.

[6] Rambaut A., Holmes E.C., O’Toole Á., Hill V., McCrone J.T., Ruis C., Du Plessis L., Pybus O.G. (2020). A dynamic nomenclature proposal for SARS-CoV-2 lineages to assist genomic epidemiology. Nat. Microbiol..

[7] Alm E., Broberg E.K., Connor T., Hodcroft E.B., Komissarov A.B., Maurer-Stroh S., Melidou A., Neher R.A., O’Toole Á., Pereyaslov D. (2020). Geographical and temporal distribution of SARS-CoV-2 clades in the WHO European Region, January to June 2020. Eurosurveillance.

[8] Garcia-Beltran W.F., Lam E.C., Denis K.S., Nitido A.D., Garcia Z.H., Hauser B.M., Feldman J., Pavlovic M.N., Gregory D.J., Poznansky M.C. (2021). Multiple SARS-CoV-2 variants escape neutralization by vaccine-induced humoral immunity. Cell.

[9] Di Caro A., Cunha F., Petrosillo N., Beeching N.J., Ergonul O., Petersen E., Koopmans M.P. (2021). Severe acute respiratory syndrome coronavirus 2 escape mutants and protective immunity from natural infections or immunizations. Clin. Microbiol. Infect..

[10] Funk T., Pharris A., Spiteri G., Bundle N., Melidou A., Carr M., Gonzalez G., Garcia-Leon A., Crispie F., O’Connor L. (2021). Characteristics of SARS-CoV-2 variants of concern B.1.1.7, B.1.351 or P.1: Data from seven EU/EEA countries, weeks 38/2020 to 10/2021. Eurosurveillance.

[11] Faria N.R., Mellan T.A., Whittaker C., Claro I.M., Candido D.D.S., Mishra S., Crispim M.A.E., Sales F.C.S., Hawryluk I., McCrone J.T. (2021). Genomics and epidemiology of the P.1 SARS-CoV-2 lineage in Manaus, Brazil. Science.

[12] Jangra S., Ye C., Rathnasinghe R., Stadlbauer D., Krammer F., Simon V., Martinez-Sobrido L., García-Sastre A., Schotsaert M., Alshammary H. (2021). SARS-CoV-2 spike E484K mutation reduces antibody neutralisation. Lancet Microbe.

[13] Tegally H., Wilkinson E., Giovanetti M., Iranzadeh A., Fonseca V., Giandhari J., Doolabh D., Pillay S., San E.J., Msomi N. (2021). Detection of a SARS-CoV-2 variant of concern in South Africa. Nat. Cell Biol..

[14] Kemp S.A., Collier D.A., Datir R.P., Ferreira I.A.T.M., Gayed S., Jahun A., Hosmillo M., Rees-Spear C., Mlcochova P., Lumb I.U. (2021). SARS-CoV-2 evolution during treatment of chronic infection. Nat. Cell Biol..

[15] Deng X., Garcia-Knight M.A., Khalid M.M., Servellita V., Wang C., Morris M.K., Sotomayor-González A., Glasner D.R., Reyes K.R., Gliwa A.S. (2021). Transmission, infectivity, and antibody neutralization of an emerging sars-cov-2 variant in california carrying a l452r spike protein mutation. medRxiv Prepr. Serv. Health Sci..

[16] Dong E., Du H., Gardner L. (2020). An interactive web-based dashboard to track COVID-19 in real time. Lancet Infect. Dis..

[17] Katoh K., Standley D.M. (2013). MAFFT Multiple Sequence Alignment Software Version 7: Improvements in Performance and Usability. Mol. Biol. Evol..

[18] De Maio N., Walker C., Borges R., Weilguny L., Slodkowicz G., Goldman N. (2021). Masking Strategies for SARS-CoV-2 Alignments. Virological.org. https://virological.org/t/masking-strategies-for-sars-cov-2-alignments/480.

[19] Kumar S., Stecher G., Li M., Knyaz C., Tamura K. (2018). MEGA X: Molecular evolutionary genetics analysis across computing platforms. Mol. Biol. Evol..

[20] Minh B.Q., Schmidt H.A., Chernomor O., Schrempf D., Woodhams M.D., von Haeseler A., Lanfear R. (2020). IQ-TREE 2: New Models and Efficient Methods for Phylogenetic Inference in the Genomic Era. Mol. Biol. Evol..

[21] Kalyaanamoorthy S., Minh B.Q., Wong T.K.F., Von Haeseler A., Jermiin L.S. (2017). ModelFinder: Fast model selection for accurate phylogenetic estimates. Nat. Methods.

[22] Letunic I., Bork P. (2019). Interactive Tree Of Life (iTOL) v4: Recent updates and new developments. Nucleic Acids Res..

[23] Rambaut A., Lam T.T., Carvalho L.M., Pybus O. (2016). Exploring the temporal structure of heterochronous sequences using TempEst (formerly Path-O-Gen). Virus Evol..

[24] Suchard M.A., Lemey P., Baele G., Ayres D.L., Drummond A.J., Rambaut A. (2018). Bayesian phylogenetic and phylodynamic data integration using BEAST 1.10. Virus Evol..

[25] Drummond A.J., Rambaut A., Shapiro B., Pybus O. (2005). Bayesian Coalescent Inference of Past Population Dynamics from Molecular Sequences. Mol. Biol. Evol..

[26] Rambaut A., Drummond A.J., Xie D., Baele G., Suchard M.A. (2018). Posterior Summarization in Bayesian Phylogenetics Using Tracer 1.7. Syst. Biol..

[27] Stadler T., Kühnert D., Bonhoeffer S., Drummond A. (2013). Birth-death skyline plot reveals temporal changes of epidemic spread in HIV and hepatitis C virus (HCV). Proc. Natl. Acad. Sci. USA.

[28] Bouckaert R., Vaughan T.G., Barido-Sottani J., Duchêne S., Fourment M., Gavryushkina A., Heled J., Jones G., Kühnert D., De Maio N. (2019). BEAST 2.5: An advanced software platform for Bayesian evolutionary analysis. PLoS Comput. Biol..

[29] Li Q., Guan X., Wu P., Wang X., Zhou L., Tong Y., Ren R., Leung K.S.M., Lau E.H.Y., Wong J.Y. (2020). Early Transmission Dynamics in Wuhan, China, of Novel Coronavirus-Infected Pneumonia. N. Engl. J. Med..

[30] Nie Q., Li X., Chen W., Liu D., Chen Y., Li H., Li D., Tian M., Tan W., Zai J. (2020). Phylogenetic and phylodynamic analyses of SARS-CoV-2. Virus Res..

[31] R Core Team (2020). R: A Language and Environment for Statistical Computing.

[32] Venables W.N., Ripley B.D. (2002). Modern Applied Statistics with s. Fourth.

[33] Wei T., Simko V., R package Corrplot: Visualization of a Correlation Matrix (2017). Version 0.84. https://github.com/taiyun/corrplot.

[34] Meyer D., Zeileis A., Hornik K. (2006). The strucplot framework: Visualizing multi-way contingency tables with VCD. J. Stat. Softw..

[35] Sironi M., Hasnain S.E., Rosenthal B., Phan T., Luciani F., Shaw M.-A., Sallum M.A., Mirhashemi M.E., Morand S., González-Candelas F. (2020). SARS-CoV-2 and COVID-19: A genetic, epidemiological, and evolutionary perspective. Infect. Genet. Evol..

[36] Parczewski M., Ciechanowicz A. (2020). Molecular epidemiology of SARS CoV-2: A review of current data on genetic variability of the virus. Pol. Arch. Intern. Med..

[37] Lai A., Bergna A., Acciarri C., Galli M., Zehender G. (2020). Early phylogenetic estimate of the effective reproduction number of SARS-CoV-2. J. Med Virol..

[38] Lai A., Bergna A., Caucci S., Clementi N., Vicenti I., Dragoni F., Cattelan A.M., Menzo S., Pan A., Callegaro A. (2020). Molecular Tracing of SARS-CoV-2 in Italy in the First Three Months of the Epidemic. Viruses.

[39] Hoffmann M., Zhang L., Krüger N., Graichen L., Kleine-Weber H., Hofmann-Winkler H., Kempf A., Nessler S., Riggert J., Winkler M.S. (2021). SARS-CoV-2 mutations acquired in mink reduce antibody-mediated neutralization. Cell Rep..

[40] Thomson E.C., Rosen L.E., Shepherd J.G., Spreafico R., Filipe A.D.S., Wojcechowskyj J.A., Davis C., Piccoli L., Pascall D.J., Dillen J. (2021). Circulating SARS-CoV-2 spike N439K variants maintain fitness while evading antibody-mediated immunity. Cell.

[41] Brejová B., Hodorová V., Boršová K., Čabanová V., Reizigová L., Paul E.D., Čekan P., Klempa B., Nosek J., Vinař T.B. 258$\Delta$, a Sars-cov-2 Variant with $\Delta$h69/$\Delta$v70 in the Spike Protein Circulating in the Czech Republic and Slovakia. https://arxiv.org/pdf/2102.04689.

[42] Zhang L., Jackson C.B., Mou H., Ojha A., Peng H., Quinlan B.D., Rangarajan E.S., Pan A., Vanderheiden A., Suthar M.S. (2020). Sars-cov-2 spike-protein d614g mutation increases virion spike density and infectivity. Nat. Commun..

[43] Yi B., Poetsch A.R., Stadtmüller M., Rost F., Winkler S., Dalpke A.H. (2020). Phylogenetic analysis of sars-cov-2 lineage development across the first and second waves in eastern Germany. bioRxiv.

