# Supplementary material for: Molecular Evolution and Epidemiological Characteristics of SARS COV-2 in (Northwestern) Poland
Source: Viruses. 2021 Jul 2;13(7):1295. doi: 10.3390/v13071295 (PMC8310356; doi:10.3390/v13071295)
Supplement: Supplementary file 1 [file viruses-13-01295-s001.zip › viruses-1246662-supplementary.pdf]

Supplementary data

Prevalence of Sars-CoV-2 variants

Based on the PANGOLIN and NEXTCLADE methods of lineage classification in the main text we presented four lineages (B.1.221; B.1.258; B.1.1.29; and B.1.1.7) which accounted for more than 10% of infections in Poland. The remaining variants were listed in Supplemental Figure 1 and Supplementary Table 2. Interestingly 67 (6.06%) genomes of PANGO lineage B.1 had different Nextstrain clade, including 54 of type 20A, 12 of type 20C, and one of strain 20B. In 61 (5.52%) isolates variant B.1.177 (20E EU1) has been identified. In the studied group (n=1106), 19 (1.72%) sequences were identified as Nextstrain 19A clade and a single isolate as 19B. This assembly of sequences belonged to the oldest phylogenetic clades, which emerged in Wuhan comprised of 13 genomes (1.18%) belonged to PANGO lineage B.40, four (0.36%) inferred as variants B, and each one (0.09%) for type B.3 and B.39. A single sequence of 19B clade was classified as PANGO A2 type.

In five investigated variants, clades from West Pomerania had significantly different proportions compared to the entire studied sample. In north-western Poland, the dominant variant was B.1.258 (20A), and an increased incidence of B.1.221 (20A) isolates was observed. In contrast, lower distribution of three lineages: B.1.1.29 (20B), B.1.1.7 (20I/501Y.V1), and B.1 (20A) were noted in West Pomerania as related to the whole country (Supplemental Figure 2A and B). Sampling for West Pomeranian province started in November 2020. As virus variants B.1.258 (20A) and B.1.221 (20A) appeared in the Polish population in November 2020 and variant B.1.1.7 (20I/501Y.V1) in late December (main Figure 1), there is no risk of overestimating samples from the rest of the national regions. The lineages B.1(20A) and B.1.1.29 (20B) covered the timeframe of a pandemic from March 2020. To exclude the possibility that frequency changes for lineages B.1.1.29 (20B) and B.1 (20A) among provinces were induced by the oversampling before November 2020, we also compared these two virus types only for the last four months of the analysis (Supplemental Figure 2B). This approach reveals that, between November 2020 and February 2021, there were no notable differences in the incidence of lineages B.1 (20A) and B.1.1.29 (20B) between the West Pomeranian region and the rest of the country.

Identified clades and Nextstrain and PANGO variants in Northwestern Poland (n=122; see main figure 2)

Clade 1 (n=34, 27.87%)

Nextrain: 20B (n=32, 26.23%%) and 20I/501Y.V1 (n=2, 1.64%)
PANGO: B.1.1.153 (n=10, 8.20%), B.1.1.277 (n=6, 4.92%), B.1.1.29 (n=4, 3.28%) B.1.170 (n=4; 3.28%), B.1.1.141 (n=3; 2.46%), and B.1.1.7 (n=2, 1.64%) were observed in this clade. In this clade, a single case (0.82%) was observed for each of B.1.1.159, B.1.1.288, B.1.1.250, B.1.1.144, and B.1.1.145 variant.

Clade 2 (n=37, 30.33%)

All of the isolates in Clade 2 were assigned to Nexstrain 20A clade and PANGO B.1.258 lineage.

Clade 3 (n=34, 27.87%)

Sequences from Clade 3 belonged to the 20A Nextstrain group and PANGO B.1.221 variant.

Identified clades and Nextstrain and PANGO variants in the entire Poland (n=1106; see main figure 3)

Clade 1 (n=500, 45.21%)
Nextrain: 20B (n=364, 32.91%), 20I/501Y.V1 (n=123, 11.12%), 20D (n=13, 1.18%)
PANGO: B.1.1.29 (n=125, 11.30%), B.1.1.7 (n=123, 11.12%), B.1.1.153 (n=55, 4.97%), B.1.1.277 (n=42, 3.80%), B.1.1.170 (n=22, 1.99%), B.1.1.279 (n=16, 1.45%), B.1.1 (n=16, 1.45%), B.1.1.219 (n=15, 1.36%), B.1.1.317 (n=12, 1.08%), B.1.1.101 (n=10, 0.90%), B.1.1.250 (n=7, 0.63%), B.1.1.44 (n=6, 0.54%), B.1.1.64 (n=5, 0.45%), B.1.1.28 (n=4, 0.36%), C.10 (n=4, 0.36%) and B.1.1.141 (n=3, 0.27%). In this clade ten cases had each of 2 (0.18%) sequences (B.1.1.10, B.1.1.119, B.1.1.130, B.1.1.159, B.1.1.171, B.1.1.189, B.1.1.297, B.1.1.239, B.1.1.307, B.1.1.67) and in sixteen cases had each of one (0.09%) sequence (B.1, B.1.1.117, B.1.1.127, B.1.1.145, B.1.1.15, B.1.1.164, B.1.1.167, B.1.1.208, B.1.1.282, B.1.1.285, B.1.1.288, B.1.1.296, B.1.1.4, B.1.1.47, B.1.1.99).

Clade 2 (n=160, 14.47%)
In the Clade 2 all genomes belonged to Nextstrain clade 20A and PANGO B.1.258 lineage.

Clade 3 (n=163, 14.74%)
For the Clade 3 all sequences correspond to Nextstrain clade 20A and PANGO B.1.221 variant.

In country-level phylogeny, seven sequences that did not follow the clade defining mutations pattern were found inside Clades 1-3 and were excluded from the analysis (main Figure 3).

Identified clades and Nextstrain and PANGO variants in the international sequences (n=376; see main figure 4).

Clade 1 (n=111, 29.52%)

Nextrain: 20B (n=91, 24.20%) and 20I/501Y.V1 (n=20, 5.32%)
PANGO: B.1.1.29 (n=33, 8.78%), B.1.1.7 (n=20, 5.32%), B.1.1.153 (n=14, 3.72%), B.1.1.170 and B.1.1.277 (n=11, 2.93%), B.1.1.141 (n=5, 1.33%), B.1.1.281 and B.1.1.250 (n=3, 0.80%), B.1.1.284 and B.1.1.219 (n=2, 0.53%). In this clade, a single case (0.27%) was observed for each of B.1.1, B.1.1.101, B.1.1.207, B.1.1.230, B.1.1.238, B.1.1.247, and B.1.1.303 variant.

The highest virus variants prevalence for Clade 1 occurred in three countries: Germany (n= 29, 7.71%), UK (n=18, 4.79%), Iceland (n=14, 3.72%)

Clade 2 (n=65, 17.29%)

Nextrain: 20A (n=65, 17.29%)
PANGO: B.1.258 (n=62, 16.49%), B.1.1.243(n=3, 0.80%)

The highest virus variants prevalence for Clade 2 occurred in three countries: Germany (n= 23, 6.12%), United Kingdom (n=16, 4.26%), and Poland (n=4, 1.06%)

Clade 3 (n=86, 28.87%)

All sequences correspond to Nextstrain clade 20A / PANGO B.1.221 variant.

The highest virus variants prevalence for Clade 3 inferred mostly around the German (n=38, 10.11%), Switzerland (n=11, 2.93%) and Norway (n=9, 2.39%) sequences.

Identified Nextstrain and PANGO clades and spike protein mutations

D614G

Substitution D614G was detected in almost every sequence (97.47%), constantly throughout the study timeframe, and only 28 cases did not possess this change. In those group 19 isolates from March and April 2020 (1.72%) correspond to Nextstrain clade 19A and PANGO variants B.40 (n=13, 1.18%), B (n=4, 0.36%), B.39 (n=1, 0.09%) and B.3 (n=1, 0.09%) and one sequence (0.09%) linked to Nextstrain clade 19B and PANGO variant A2. The remaining eight isolates were collected from September 2020 to February 2021. They belonged to different clades, including two (0.18%) variants for 20A (PANGO lineage B.1 and B.1.258), two variants for 20B (PANGO lineage B.1.1.29 and B.1.1.250), and each one for 20C (PANGO B.1), 20G (PANGO B.1.2), 20E EU1 (PANGO B.1.177), and 20I/501Y.V1 (PANGO B.1.1.7).

ΔH69V70

This mutation was present in Clade 1 and Clade 2. For Clade 1, the number of isolates with this mutation was 122 (11.03%), all for lineage B.1.1.7 (Nextstrain 20I/501Y.V1), while for Clade 2, 157 (14.19%) of the sequence belonged entirely to variant B.1.258 (20A). The first isolate was registered on the 2nd November 2020, and the last on 23rd February 2021.

P681H

This variant was noted in 220 (19.89%) samples and arose between the end of October 2020 till February 2021. Except for five (0.45%) sequences (of PANGO lineage B.1.258, Nextstrain 20A) that did not match our clades, all genomes with P681H substitution were associated with Clade 1 and was found in great types of virus. In this group the most prevalent isolates (n=123, 11,12%) belonged to PANGO variant B.1.1.7 (Nextstrain 20I/501Y.V1), followed by 57 (5.15%) cases of B.1.1.29 (20B) lineage. Five (0.45%) sequences that possessed S:P681H mutation were observed for lineage B.1.1.64 (20B), three for B.1.1.44 (20B), two (0.18%) for each of B.1.1 (20B), B.1.10 (20B), B.1.1.119 (20B), B.1.1.1.89 (20B), and B.1.1.239 (20B). Only one genome (0.09%) with the S:P681H substitution was noted for variants B.1.1.117, B.1.1.127, B.1.1.130, B.1.1.145, B.1.1.170, B.1.1.171, B.1.1.250, B.1.1.279, B.1.1.282, B.1.1.285, B.1.1.67, B.1 (all Nextstrain clade 20B) and one B.1 (20C).

N439K

This substitution was the fourth dominant mutation in Poland and became prevalent between November 2020 and February 2021. Among 170 (15.37%) sequences, all belonged to strain B.1.258 (20A), and 160 (14.47%) were connected to our Clade 2.

S98F

The S98F mutation was observed in 15.10% cases (n=167), appeared in mid-November 2020 and was continuously observed until February 2021. Almost every sequence (n=163, 14.74%) with this mutation was defined as Clade 3 and related to PANGO variant B.1.221 (Nextstrain clade20A). Outside Clade 3 recorded three isolates that possessed S:S98F substitution. One belonged to B.1.221 (20A) variant, the rest isolates referred to type B.1 (20A) (n=2, 0.18%) and single to B.1.258 (20A) type.

T716I, S982A, delY144, A570D, N501Y, and D1118H

Those six key mutations occurred in Poland in late December and were related to the rapid expansion of B.1.1.7 (20I/501Y.V1) virus type continuously to the end of February 2021. The six mutations with prevalence >5% were: T716I (n=124, 12.21%), S982A (n=123, 11.12%), delY144 (n=123, 11.21%), A570D (n=122, 11.03%), N501Y (n=122, 11.03%) and D1118H (n=121, 10.94%). These were strongly correlated to B.1.1.7 (20I/501Y.V1) variant of concern (main Figure 6). Only one substitution T716I and one deletion delY144 also belonged to different virus variant B1 (20C) and B.1.1.170 (20B), respectively.

A222V

The last top frequency mutation was A222V found in 71 (6.42%) cases. All sequences were PANGO lineage B.1.1.177 (20E EU1) except two genomes being B.1.160 (20A EU2) variants. Substitution A222V was first observed in mid-November 2020 and until February 2021. The group of isolates that bearing A222V mutation was located outside the pre-defined Clades. The remaining identified mutations in Spike protein were placed in Supplementary Table 3.

Temporal trends for Spike mutation frequency in non-B.1.1.7 variants

The proportion of the P681H substitution increased steadily from 0.88% in November to 18.42% in February (OR: 2.04, 95% CI 1.15-2.77, p<0.0001) (see Supplementary Table 4 and Supplemental Figure 4). The frequency of mutation A222V increased significantly from 7.02% in November to 15.79% (OR: 1.35, 95% CI 1.00-1.84, p=0.047) in February among individuals diagnosed with non-B.1.1.7 virus. Additionally, the proportion of infections other than the B.1.1.7 variants with detected S98F mutation increased from 6.14% in November to 22.36% in February (OR: 1.57, 95% CI 1.27-1.93, p<0.0001). Among non-B.1.1.7 variant infected individuals, the frequency of cases with deletion lH69V70 decreased over time from 26.31% in November to 21.05% in February (OR: 0.78, 95% CI 0.64-0.95, p=0.014).

Table S1. Estimates of average evolutionary divergence over West Pomeranian sequence pairs within and between Clades. The number of base substitutions per site from averaging over all sequence pairs within (and between) each Clade are shown. Analyses were conducted using the Kimura 2-parameter model. The rate variation among sites was modeled with a gamma distribution. Evolutionary analyses were conducted in MEGA X.

	Intra-divergence			Inter- divergence	
	d	sd		d	sd
Clade 1	0,000983	8,88996E-05	Clade 1-2	0,001376	0,000151
Clade 2	0,000509	5,01419E-05	Clade 1-3	0,001266	0,000142
Clade 3	0,000482	5,12151E-05	Clade 2-3	0,001303	0,000172

Table S2. Differences in Sars-CoV-2 variants prevalence by analyzed region. Statistical differences calculated for the regional variant distribution versus the entire sample of 1106 patients. Calculated by χ2 test, for samples <5 Fisher exact test was used.

PANGO Variants	West Pomerania		Poland		Other PANGO Variants	West Pomerania		Poland	
All	n=122	p	n=1106	p	All	n=9	p	N=99	p
B.1.221	33 (27.05%)	0.00015	170 (15.37%)	ref.	B.1.1.250	1 (0.82%)	0.609	7 (0.63%)	ref.
B.1.258	38 (31.15%)	>0.00001	166 (15.01%)		B.1.1.44	1 (0.82%)	0.560	6 (0.54%)	
B.1.1.29	4 (3.28%)	0.00258	127 (11.48%)		B.1.1.64; B.1.1 B.1.223	0		3 (0.27%)	
B.1.1.7	2 (1.64%)	0.00041	123 (11.12%)		B.1.510; B; B.1.1.28 B.1.98; C.10	0		4 (0.36%)	
B.1	1 (0.82%)	0.0046	67 (6.06%)		B.1.389	1 (0.82%)	0.443	4 (0.36%)	
B.1.177	7 (5.74%)	0.909	61 (5.52%)		B.1.1.141	3 (2.46%)	0.0204	3 (0.27%)	
B.1.1.153	10 (8.20%)	0.825	55 (4.97%)		B.1.1.159	1 (0.82%)	0.296	2 (0.18%)	
B.1.1.277	6 (4.92%)	0.492	42 (3.80%)		B.1.288	1 (0.82%)	0.209	1 (0.18%)	
B.1.213	0		32 (2.89%)		B.1.1.10; B.1.1.119; B.1.1.130; B.1.1.171; B.1.1.189; B.1.1.239; B.1.1.297; B.1.1.307; B.1.1.67; B.1.13; B.1.1.343; B.1.91	0		2 (0.18%)	
B.1.1.170	4 (3.28%)	0.292	22 (1.99%)						
B.1.2	5 (4.10%)	0.077	22 (1.99%)						
B.1.1.279	0		16 (1.45%)						
B.1.88	3 (2.46%)	0.41	16 (1.45%)						
B.1.1.219	0		15 (1.36%)						
B.1.160	0		15 (1.36%)						
B.40	0		13 (1.18%)						
B.1.1.317	0		12 (1.08%)		A.2; B.1.1.117; B.1.1.127; B.1.1.15; B.1.1.164; B.1.1.167; B.1.1.208; B.1.1.282; B.1.1.285; B.1.1.296; B.1.1.4; B.1.1.47; B.1.1.99; B.1.153; B.3; B.39	0		1 (0.18%)	
B.1.187	0		12 (1.08%)						
B.1.1.1	0		11 (0.99%)						
B.1.1.101	0		10 (0.90%)						

Other	9 (7.38%)	0.518	99 (8.95%)					

Table S3. Differences in mutations of Sars-CoV-2 spike protein prevalence by analyzed region. Statistical differences calculated for the regional mutation distribution versus the entire sample of 1106 patients. Calculated by χ^2 test, for samples <5 Fisher exact test was used.

Mutations	West Pomerania	p	Poland	p
S:D614G	119 (97.54%)	0.687	1092 (98.73%)	ref.
S:delH69V70	38 (31.15%)	0.551	281 (25.23%)	
S:P681H	10 (8.20%)	0.0003	220 (19.89%)	
S:N439K	36 (29.51%)	0.0106	170 (15.37%)	
S:S98F	34 (27.87%)	0.0261	165 (14.92%)	
S:T716I	2 (1.64%)	>0.0001	124 (11.21%)	
S:S982A	2 (1.64%)	>0.0001	123 (11.12%)	
S:delY144	2 (1.64%)	>0.0001	123 (11.12%)	
S:A570D	2 (1.64%)	>0.0001	122 (11.03%)	
S:N501Y	2 (1.64%)	>0.0001	122 (11.03%)	
S:D1118H	2 (1.64%)	>0.0001	121 (10.94%)	
S:A222V	7 (5.74%)	0.548	71 (6.42%)	
S:A626S	6 (4.92%)	0.272	30 (2.71%)	
S:L18F	1 (0.82%)	0.384	25 (2.26%)	
S:S477N	1 (0.82%)	0.506	23 (2.08%)	
S:T95I	0		10 (0.90%)	
S:L5F	0		8 (0.72%)	
S:Q667H	0		7 (0.63%)	
S:D138Y	0		6 (0.54%)	
S:Y453F	0		3 (0.27%)	
S:A701V	0		2 (0.18%)	
S:H655Y	2 (1.64%)	0.062	2 (0.18%)	
S:T1027I	1 (0.82%)	0.209	1 (0.09%)	

Table S4. Time Trends for missense mutations in Spike Protein in non-B.1.1.7 variant infected population

Spike mutation	non-B.1.1.7 (20H/501Y.V2) n=983				OR	95% CI	p
	Nov 20	Dec 20	Jan 21	Feb 21			
P681H	1 (0.88%)	14 (8.64%)	67 (16.63%)	14 (18.42%)	2.04	1.15-277	<0.0001
A222V	8 (7.02%)	12 (7.41%)	39 (9.68%)	12 (15.79%)	1.35	1.00-1.84	0.047
S98F	7 (6.14%)	32 (19.75%)	111 (27.54)	17 (22.36%)	1.57	1.27-1.93	<0.0001
ΔH6970V	30 (26.31%)	46 (28.39%)	67 (16.63%)	16 (21.05%)	0.78	0.64-0.95	0.014

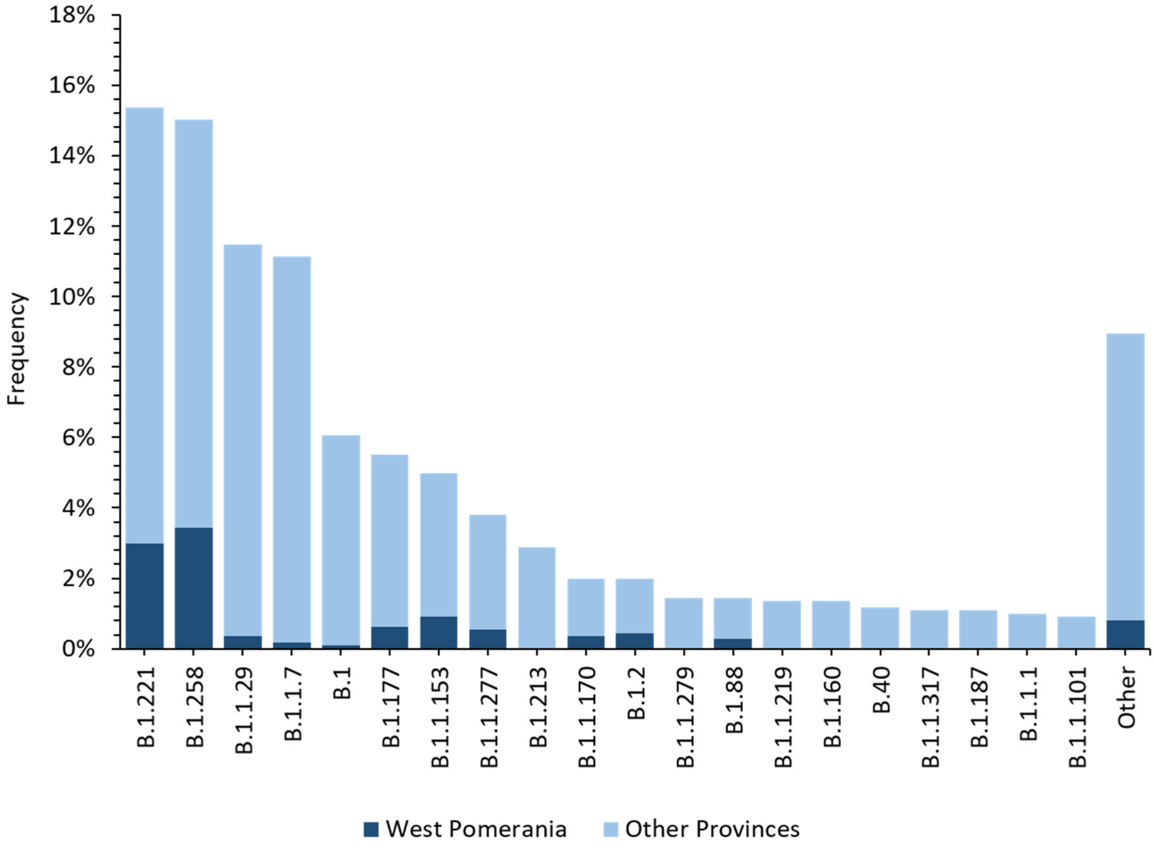

Figure S1. Prevalence of main SARS-CoV-2 lineages in Poland estimated from local the whole-genome sequences and GISAID sequences (data analyzed for March 2020-February 2021). Variant codes based on PANGO classification.

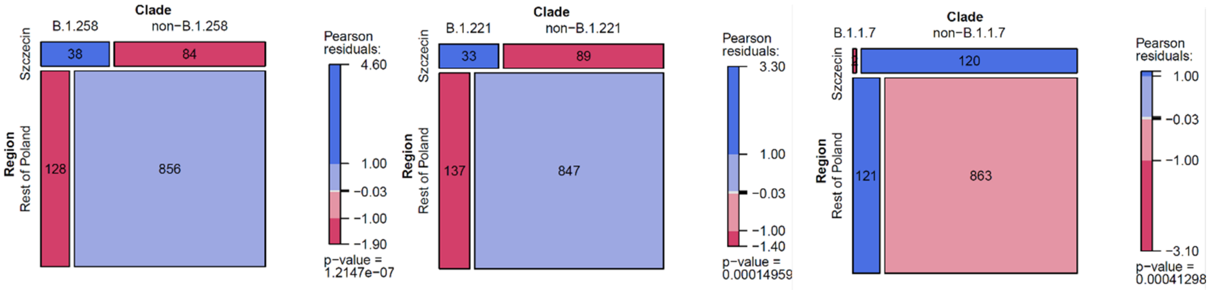

Figure S2A. Two-way contingency plots of differences in Sars-CoV-2 variant prevalence by analyzed region. Plots represent the frequency of the Sars-Cov-2 majority in Poland from November 2020 to February 2021. Statistical differences were calculated for the regional variant form distribution versus the entire sample of 1106 patients. Variants that constitute at least 5% of recorded cases and had notable differences were presented. Calculated by X2 test, for samples <5 Fisher exact test was used. Szczecin stands as the capital of the West Pomerania region.

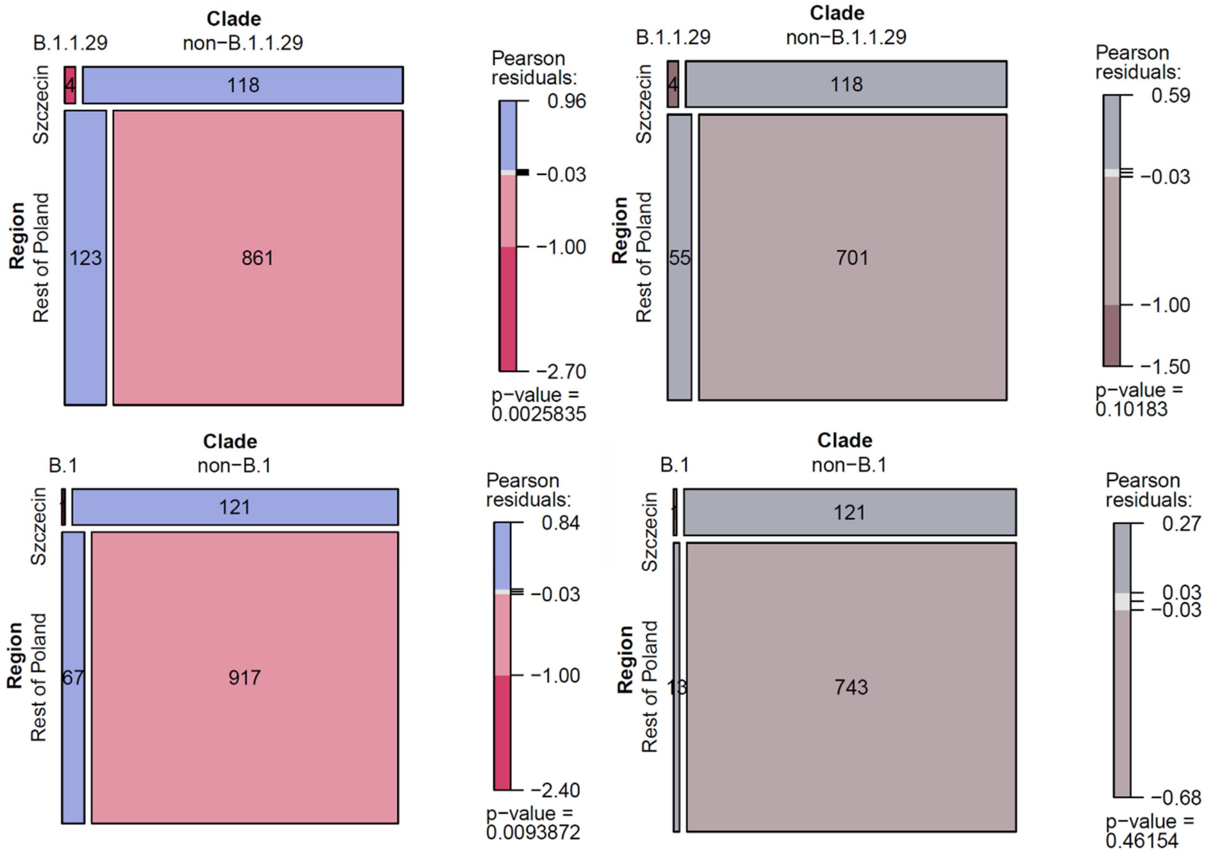

Figure S2B. Two-way contingency plots for differences in Sars-CoV-2 variant prevalence by analyzed region. Plots on the left side span the entire time of the pandemic from March 2020 to February 2021. Plots on the right represent the frequency of Sars-Cov-2 prevalence in Poland from November 2020 to February 2021. Statistical differences were calculated for the regional variant form distribution versus the entire sample of 1106 patients. Variants constitute at least 5% of recorded cases and had notable differences.

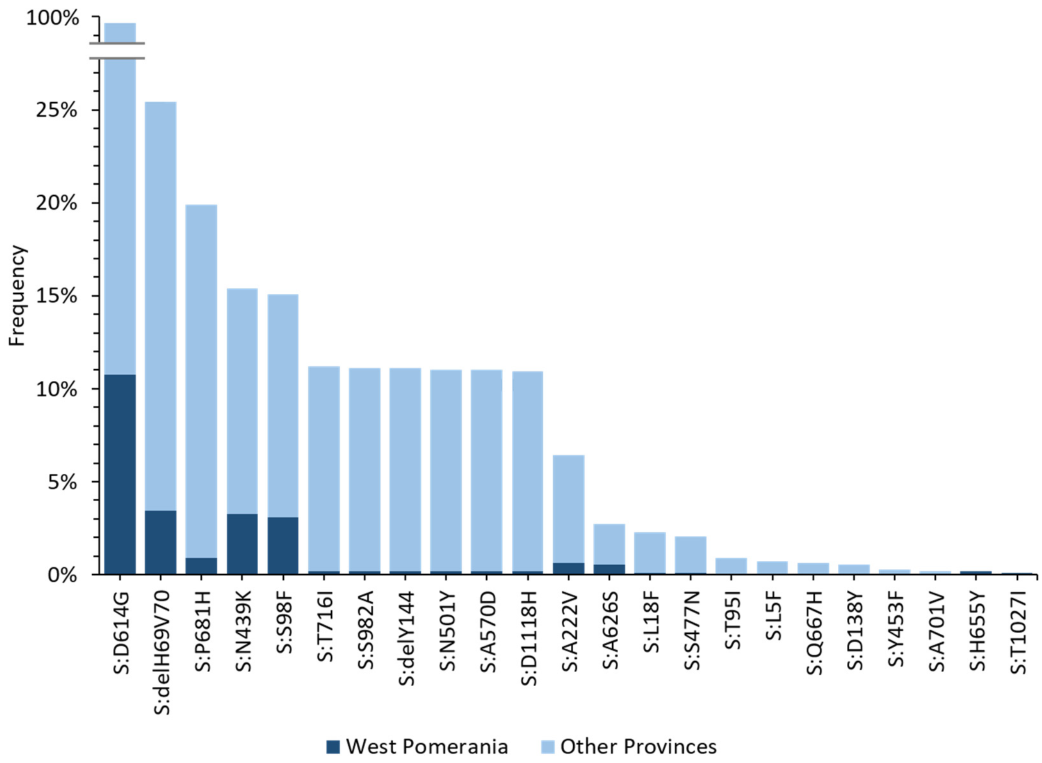

Figure S3. Frequency of major Spike mutation identified in Polish Sars-CoV-2 strains

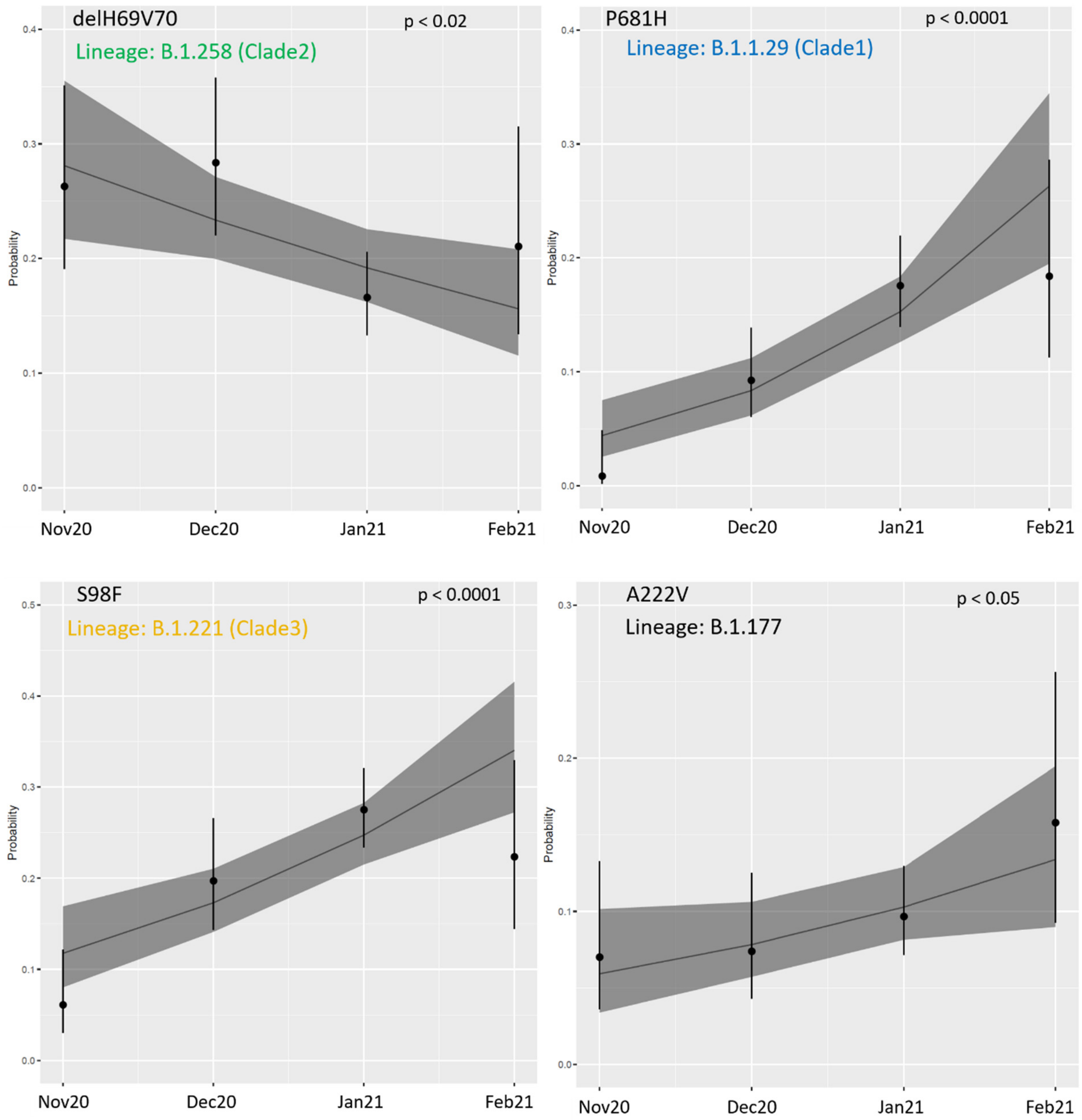

Figure S4. Logistic regression estimates for time trends between Nov 2020 and Feb 2021 for Spike protein mutations identified in Polish isolates excluding sequences with B.1.1.7 variant.

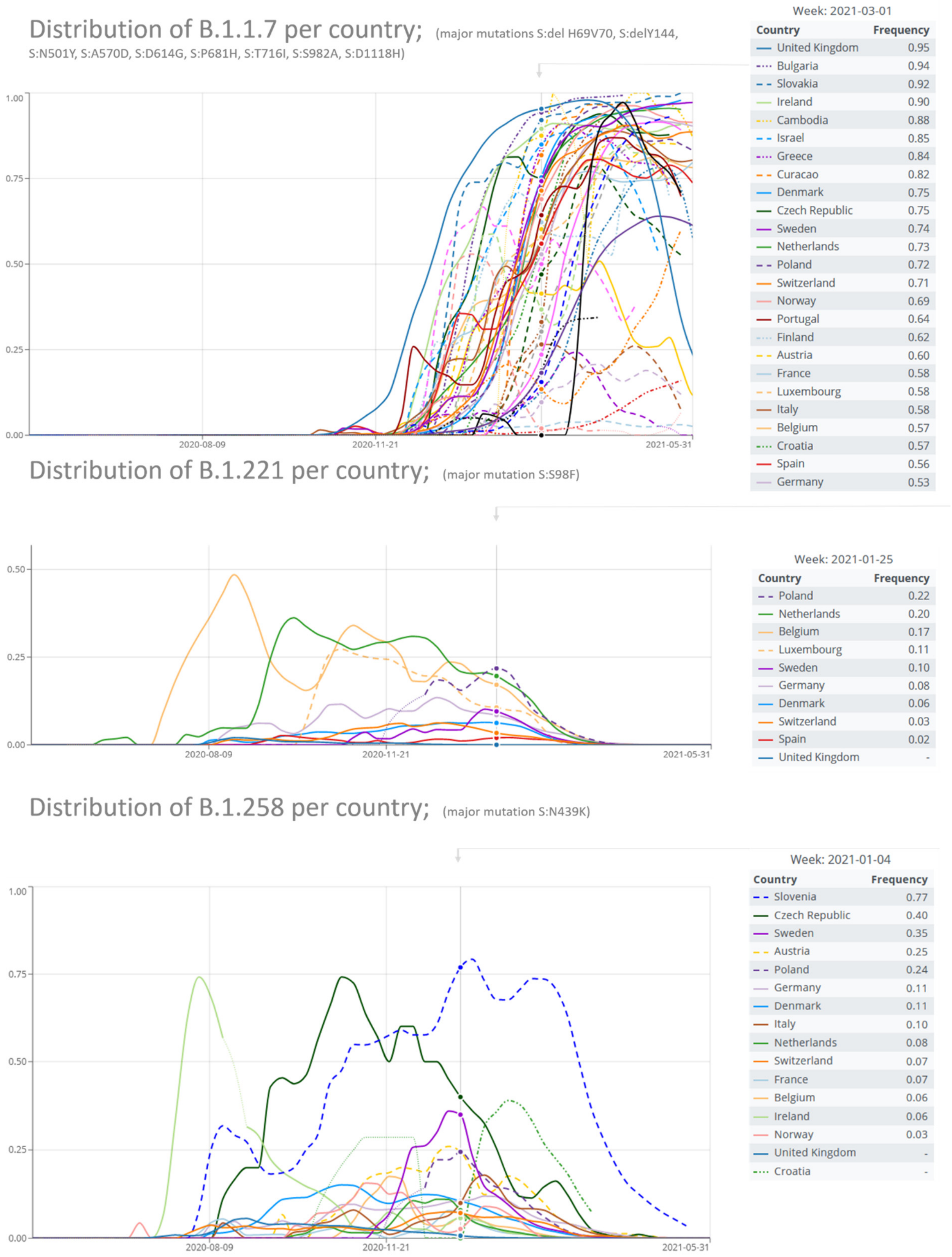

Figure S5. Local lineage dynamics for B.1.1.7 (Clade 1), B.1.221 (Clade 3) and B.1.258 (Clade 2) virus variants. For polish sequences, the date of the highest frequency for each lineage was marked. Based on https://covariants.org (accession on 22 June 2021)
